# Timeless spaces: Field experiments in the physiological study of circadian rhythms, 1938–1963

**DOI:** 10.1007/s40656-023-00571-w

**Published:** 2023-04-19

**Authors:** Kristin D. Hussey

**Affiliations:** Medical Museion and the Novo Nordisk Foundation Center for Basic Metabolic Research (CBMR), University of Copenhagen, Copenhagen, Denmark

**Keywords:** Circadian rhythm, Physiology, Experiment, Cold War, Fieldwork, Temporality, Chronobiology

## Abstract

In the middle of the twentieth century, physiologists interested in human biological rhythms undertook a series of field experiments in natural spaces that they believed could closely approximate conditions of biological timelessness. With the field of rhythms research was still largely on the fringes of the life sciences, natural spaces seemed to offer unique research opportunities beyond what was available to physiologists in laboratory spaces. In particular, subterranean caves and the High Arctic became archetypal ‘natural laboratories’ for the study of human circadian (daily) rhythms. This paper is explores the field experiments which occurred in these ‘timeless spaces’. It considers how scientists understood these natural spaces as suitably ‘timeless’ for studying circadian rhythms and what their experimental practices can tell us about contemporary physiological notions of biological time, especially its relationship to ‘environmentality’ (Formosinho et al. in Stud History Philos Sci 91:148–158, 2022). In so doing, this paper adds to a growing literature on the interrelationship of field sites by demonstrating the ways that caves and the Arctic were connected by rhythms scientists. Finally, it will explore how the use of these particular spaces were not just scientific but also political – leveraging growing Cold War anxieties about nuclear fallout and the space race to bring greater prestige and funding to the study of circadian rhythms in its early years.

## Introduction

In 1969, the British biologist Philip Corbet wrote a short commentary in *Nature* on ‘The Arctic as an environment for research on rhythms’. While a growing number of physiologists working on circadian rhythms in humans had been attracted by the unique environmental conditions of the Arctic summer, Corbet worried that such experiments were fundamentally flawed. These studies made the assumption that ‘the Arctic during the summer … lacks obvious time cues’ and therefore could be considered a biologically timeless space (Corbet, 1969, p. 392). Corbet was quick to dispel this belief – he asserted that it was perfectly possible to tell time by the sun in the Arctic summer, to say nothing of the associated daily changes in ambient temperature. ‘Uniformity of light intensity and temperature… can be secured more effectively and cheaply in other ways,’ he concluded. To this point, Corbet drew attention to an exciting new series of experiments taking place a disused World War II bunker in Andechs, Germany.^1^ A few years later, the doyen of the emerging science of chronobiology Franz Halberg (1919–2013) and his collaborators (Simpson et al., 1973) countered Corbet’s assertion, arguing that Arctic experiments were superior to a laboratory environment because subjects were able to live a normal routine for an extended period rather than being isolated in a bunker or metabolic room. For Halberg, polar spaces were nothing short of a ‘natural laboratory’ for the study of biological rhythms.

What made something a good space for studying human circadian rhythms in the mid-twentieth century? Was it a laboratory, a bunker, a cave, or an Arctic hut? As Corbet’s commentary indicates, by about the year 1970, it was felt that rigorous experiments into rhythmicity needed to occur in specially-designed laboratory spaces – away from the possible fluctuations of temperature, light and social cues of the field. But, in the middle third of the twentieth century, field experiments offered physiologists much that the laboratory could not. In the darkness and isolation of subterranean caves, or the brightness of an Arctic summer, scientists carried out experiments in artificially modifying daily rhythms as a way of teasing out their underlying periodicity and mechanisms. Research participants, who were often the scientists themselves, lived on modified short (21- or 22-h) or long (27- or 28-h) days as a way of disrupting the body’s 24-h rhythmicity. Today, this practice would be referred to as a ‘forced desychrony protocol’ – breaking the body's adherence to clock time and allowing its inherent biological timing to take over, a phenomenon called ‘free running’ (‘Forced Desychrony’, 2009). Such protocols rely on removing a biological system (human or otherwise) from the time cues (or *zeitgebers*) which synchronize the body to its environment. What exactly these time cues are and which are more important in terms of regulating what has been colloquially called the ‘body clock’, was (and remains) a point of scientific contention. Looking more closely at these natural spaces and the actual work of designing and carrying out such experiments within them has much to tell us about mid-twentieth century scientific ideas about biological time (Rheinberger, 2002; Wellmann, 2017; Hopwood et al., 2021; Shackleford, 2022a, 2022b).

This paper will consider a series of field experiments in human circadian rhythms that occurred in cave and Arctic spaces between 1938 and 1963. Subterranean caves, like the High Arctic, offered a natural environment where research subjects could be removed from both the 24-h solar light-dark cycle and the rhythms of society. The study starts with University of Chicago physiologist Nathaniel Kleitman’s famous month-long stay in Kentucky’s Mammoth Cave in 1938, and ends in 1963 with British physiologist John Mills’ study in Stump Cross Caverns in the Yorkshire Dales. In the intervening years, it tracks the growing prominence of the Norwegian High Arctic as a site of chronobiological research through experiments by Cambridge physiologist Mary C. Lobban and again, Nathaniel Kleitman. It will demonstrate the ways in which these two experimental spaces were in constant conversation both rhetorically and scientifically. In particular, I want to argue that these spaces were conceived of as superior to the laboratory. At the same time, these natural spaces facilitated longer and more ambitious experiments than were possible in a lab setting. I focus on *why* these experimenters selected these particular spaces for their work – what made certain natural locations suitably ‘timeless’ for rhythms research? How did these scientists exploit the unique characteristics of these spaces in their experimental protocols? And what can such experiments tell us about the fluid interface between body and environment, or what a group of researchers have recently termed ‘environmentality’ (Formosinho et al., 2022)?

The field of chronobiology emerged as a distinct discipline of the life sciences in the 1960s. Its consolidation from a group of disparate and highly interdisciplinary researchers to a recognized field of study within biology is generally linked by historians to the landmark Cold Spring Harbour Symposium on ‘Biological Clocks’ held in 1960 (Cambrosio & Keating, 1983; Ramji, 2008; Roenneberg & Merrow, 2005; Shackelford, 2020). The term ‘circadian rhythm’ was coined a year earlier by Franz Halberg (1959) to refer to the body’s daily, roughly 24-h physiological rhythms. But of course, before the phrase came into scientific parlance, researchers had been interested in rhythmic oscillations in humans and animals for centuries (Shackelford, 2022a). In what is widely considered the first experiment into biological rhythms, eighteenth-century French astronomer Jean-Jacques d’Ortous de Mairan (Mairan, 1729) placed a mimosa plant in a box to see if it would still open and close its leaves without exposure to the light–dark cycle. Scientific interest in rhythms picked up pace in the late nineteenth century with physiological and medical research into body temperature and sleep deprivation in humans, and diurnal movements and activity patterns in plants, insects and animals (Allbutt, 1870; Darwin & Darwin, 1880; Laycock, 1860; Manacéïne, 1894). By the middle part of the twentieth century, researchers were increasingly focused on unravelling whether these rhythms were a response to environmental conditions or independent from them. The ‘endogenous vs. exogenous’ debate would divide the rhythms community for decades to come, becoming particularly heated in the 1950s and 1960s (Shackelford, 2020). Much of this work centred on identifying which environmental factors might influence rhythmicity – a concept which would come to be known as a ‘zeitgeber’ or a time cue that fine tunes (or ‘entrains’) the body clock to its milieu (Aschoff, 1960). Therefore, for a space to be well suited to rhythmic research, it had to be ‘timeless’ in so far as it controlled for or completely removed known environmental time cues.^2^

Thinking about field sites within chronobiology connects to a growing literature within the history of science on field experiments and their relationship to the laboratory. In recent years, scholars have started to challenge the perceived dichotomy between lab and field – demonstrating the ways that they are closely connected (De Bont, 2015; Heggie, 2019; Kohler, 2002a, 2002b; Strasser, 2018). Influenced by geographers, historians have started to delve into the specific contexts of what have been variously called field sites, laboratories, stations, and natural laboratories (Bigg et al., 2009; Naylor, 2005; Shapin, 1998; Vetter, 2012). As Robert Kohler (2002a, p. 473) famously exhorted, ‘We need to take a view of labs that includes their natural settings. We must open laboratory windows and walk through doors, and observe how they are sited and what their settings do.’ In this paper, I want to follow historian of physiology Vanessa Heggie (2016, p. 812) who argues that we need to ‘find both the unique and generalizable properties of scientific spaces’ by delving into the specificities of field sites and the practices carried out in them. In her work on Arctic huts and high altitude laboratories, Heggie (2019) has demonstrated the utility of exploring how different field sites interact with *each other* rather than always centring on the lab. I want to emphasize the connections between timeless field sites – in terms of people, ideas, and representational power. Both subterranean caves and the High Arctic acted as ‘mimetic places’ (Gieryn, 2002) for early chronobiology researchers – becoming instruments of science, uniquely placed to unravel the underlying mechanisms of human circadian rhythms. In the context of Cold War science, these field sites took on a greater political importance, which in the context of nuclear war and the space race, was rhetorically useful in promoting the relevance of circadian science to potential funders. Finally, this paper will make a crucial contribution to the currently limited critical historiography of human chronobiological research – differentiating itself both in its focus on the earlier part of the twentieth century and its prioritization of field sites over laboratory experiments and institutional contexts (Cambrosio & Keating, 1983; Ramji, 2008).^3^

## Into the cave

The cave is a place with strong associations with the evolution of the human species. To go ‘back’ to the cave is like a return to the beginning of time itself. Caves are also unique physical environments – constantly dark, humid and cold, they are fraught with physical dangers and discomforts for human visitors. Experiments in caves have taken on a central importance within the histories of sleep research and chronobiology because the unique environmental conditions they provide to experiments conducted within them, as well as the public interest that has been associated with these studies. Kleitman’s iconic 1938 experiment in Mammoth Cave is chief among these – with historians of sleep research noting how this crucial moment of media interest helped to popularize the emerging scientific field (Kroker, 2007; Wolf-Meyer, 2013). In this section, I am interested in how the subterranean cave was understood as a timeless research environment for the study of human circadian rhythms. I will do this by focusing three mid-twentieth century cave experiments: Kleitman in Mammoth Cave, French speleologist Michel Siffre in Scarcasson in 1962, and British physiologist John Mills’ research on record-breaker Geoffrey Workman in Stump Cross Cavern in 1963. Interrogating *why* experimenters selected these spaces for their work and closely attending to the experimental practices which occurred within them reveals an insight into contemporary understandings of the environmental nature of biological time cues.

Jewish-Russian refuge Nathaniel Kleitman (1895–1999) began his work on sleep and wakefulness at the University of Chicago’s Department of Physiology in the 1920s – originally working on sleep deprivation, and later becoming interested in the modifiability of daily cycles of sleep and wake. He was especially concerned about whether the 24-h sleep–wake cycle was a response to ‘conditions of existence’ or if it was intrinsic to the human body (Kleitman, 1939, p. 253). Kleitman and his colleagues were themselves the subjects for these experiments – sleeping every 48 h or every 12 h, and tracking their body temperature curve to see if it maintained its 24-h rhythmicity. If it was possible to modify the daily temperature curve by wilfully changing sleep patterns, this would provide crucial evidence that circadian cycles like sleep were a response to the environment and social convention. However, Kleitman struggled to find any temperature curve adjustment in his Chicago experiments – a fact which he attributed to the limits of his laboratory research environment. While Kleitman is credited with presiding over one of the earliest ‘sleep labs’, his early research space was in essence a single cot within the existing physiology laboratory. It was designed more for surgical experimentation on animals than the isolated, ward-like environments of later sleep laboratories (Kroker, 2007). There was no ability to control light from the windows, noise from the corridors or fluctuations of environmental temperature, to say nothing of more esoteric potential zeitgebers like ambient electricity or radio waves. As Kleitman’s protégé William Dement recalled in his *The* *Promise of Sleep* (1999, p. 31), most of the modified time experiments were done by participants (typically Kleitman’s students) who were still living ‘normal’ lives – going to work and sleeping at home with their families. The experimental limitation of zeitgebers was much more achievable at the scale of plants, flies, and mice than human research subjects. But it was precisely this challenge that Kleitman hoped to address by moving his work from the lab to the field.

Sunday periodical *The American Weekly’s* 1939 review of Kleitman’s research provides a helpful entrée to the aims and methods of the Mammoth Cave experiment:

‘Dr Nathaniel Kleitman, Associate Professor of Physiology at the University of Chicago, has been experimenting with the mystery of sleep for 15 years. But in all that time he could not get hold of what might be called a pure and unadulterated sample of slumber because nowhere on the face of the earth existed a sound-proof, light-proof, temperature-proof and visitor proof building where the world would let his sleepers alone. So he has to take his beds, with their instruments, recording every move of a sleeper, down under the earth to one of the chambers of Mammoth Cave. There the darkness is an unvarying 100 percent throughout the 24 hours, the temperature remains at exactly 54 degrees Fahrenheit whether it is noon or night, no football, no honk or motor horn, no radio, and not even thunder can penetrate…One hour of the 24 is precisely like any of the others, therefore he could ignore even the sun, turning night to day, and, what was more important, declare a 28-hour day, a six day week, and see if his sleeping mechanism would accept the novelty or go on strike’. (*American Weekly*, 1939)

**Fig. 1:**
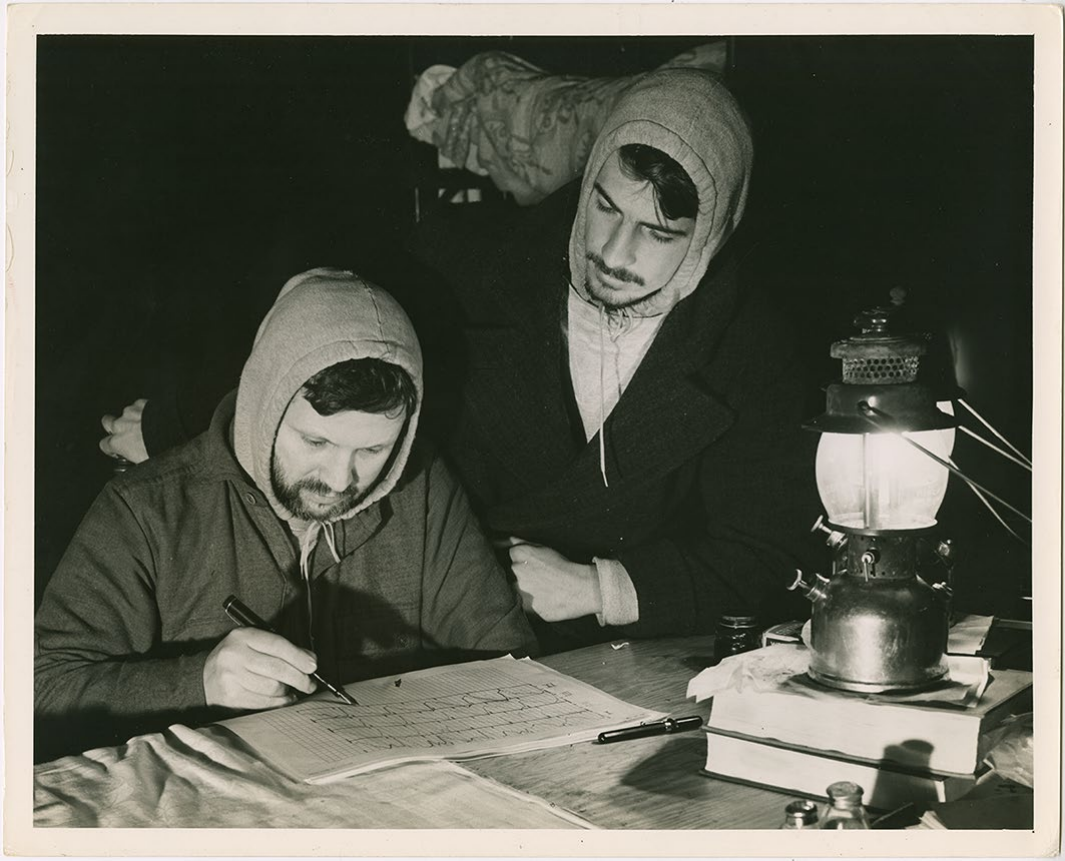
Nathaniel Kleitman and Bruce Richardson transcribing motility records in Mammoth Cave, 1938. Mammoth Cave/National Parks Service. Courtesy of the Hanna Holborn Gray Special Collections Research Center

The Mammoth Cave experiment aimed to test whether it was possible to modify the diurnal body rhythm temperature in two subjects – Kleitman himself and a graduate student assistant named Bruce Richardson. To do so, Kleitman needed a space away from what he perceived to be the most important environmental time cues – light, temperature, and ‘society’ in the broad sense of daily social rhythms including traffic noise, work patterns, and food availability. The experimental protocol consisted of living on an experimental 28-h day, with the researchers kept to schedule by an alarm clock. Kleitman provided strict instructions to the nearby Mammoth Cave hotel for the provision of two meals a day – with delivery times based on ‘surface time’ but modified to their adjusted sleep–wake cycles. For example, a first meal might be served at 1 pm, 5 pm or 9am, depending on the experimental day. These visits from the hotel staff were the researchers' only physical contact during their 32-day stay, although a messenger also relayed letters between the researchers and the outside world. Otherwise, the two men spent their time sleeping and waking on the artificial schedule – reading, playing bridge, and transcribing motility recordings (Fig. 1). The only equipment they brought with them were oral thermometers for temperature measurements taken every two hours, sleep motility readers attached to their beds (another key interest of Kleitman’s) and the alarm clock (Kleitman, 1939, p. 260). Electric lights and camping lamps allowed the men to see in the dark cave during their experimental ‘days’ – turned on and off by the researchers themselves. At the end of the experiment, Kleitman determined that his temperature rhythms had stayed essentially the same but Richardson’s had moved towards the new 28-h schedule. His conclusion was that patterns of sleeping and waking were a ‘habit’ that could be actively shifted – although how well depended on factors including age. The experiment bolstered Kleitman’s (1964) argument that ‘wakefulness of choice’, to have control over the timing of sleeping and being awake, was an evolutionary trait that set humans apart from lower animals.

Kleitman and Richardson’s stay in Mammoth Cave was relatively comfortable – the men remarking to journalists that the only drawback of their subterranean life was a ‘dampness to the sheets’ and the lack of another couple for bridge (*Chicago Sunday Tribune*, 1938). But they had the benefit of carrying out their research in a cave which was also a popular tourist site – equipped with electricity, beds, and the infrastructure of the hotel catering team. French speleologist Michel Siffre (b. 1939) had a very different experience as he travelled down into Scarasson Cavern in the Ligurian Alps in the summer of 1962. Scarasson was an unexplored glacial cavern with an internal temperature constantly below freezing and a serious risk of rock slides. While Kleitman was keen to point out in media interviews that his experiment was not a ‘show of endurance, perseverance or will power’ (quoted in Wolf-Meyer, 2013, p. 101), Siffre’s two-month stay in the cave was explicitly conceived of as a test of the body’s ability to survive in adverse circumstances; what he later dubbed in his book ‘a survival experiment’. Siffre was steeped in a tradition of adventurous exploration, and by the young age of 23 had conceived the idea of carrying out the longest underground stay ever recorded. He aimed to ‘extend the physiological limits set for human endurance’ (1965, p. 19). Siffre believed that extended exposure to cold, damp, and isolated conditions could have a relevance for soldiers and pilots who became stranded. Within this interest in adaptation to an isolated environment, Siffre also wanted to explore the psychological and physiological effects of living without any time cues. ‘I wanted to investigate time – that most inapprehensible and irreversible thing. I wanted to investigate that notion of time which has haunted humanity since its beginning’ (Siffre, 1965, p. 25). After sixty-three days underground, Siffre ‘lost’ two weeks of time as his body began to ‘free run’ – sliding in time, sleeping at progressively later and later hours, and eventually losing entire days. The cave was integral to Siffre’s approach –he took care to remove any possible interference or influence from the outside world, even taking all of his food and water down with him, and using a one-way telephone system to allow daily check-ins with colleagues above ground. His collaborators were under strict instructions to not give him any impression of the outside time until the experiment was over – which, when the time arrived, was a complete shock to Siffre.

Siffre’s cave camp was much more akin to a remote Antarctic station than to Kleitman and Richardson’s relatively comfortable camping arrangement on ‘Audubon Avenue’. Indeed, Siffre likened his experience to Admiral Richard Byrd’s infamous overwinter stay in Little America in 1934 – and equally fraught with danger (Siffre, 1965, p. 102). The tent was a fire risk, the butane stove could poison a close atmosphere, and the cold left Siffre on the brink of hypothermia. His attempts at tracking his body temperature were almost completely useless, as his thermometer would not register readings above 97F (Siffre, 1965, p. 97). However, he had more luck with tracking his changing sleep regime. Unlike Kleitman who imposed a 28-h rhythm, Siffre simply followed his own internal desire to sleep and wake. In this sense, Siffre’s approach was more like experiments done in the Andechs bunker to try and determine the intrinsic period of the body clock through the self-selection of a light–dark cycle. In his estimation, he experienced forty-two ‘physiological days’ compared to fifty-eight day-night cycles above ground. Siffre would go on to carry out and oversee numerous cave experiments around the world across the twentieth century – pushing for longer and longer amounts of time spent underground. These experiments hold a contested place in the history of chronobiology. It would be fair to say that Siffre, like many other experimenters working in natural spaces, carried with him what Richard Powell (2007, p. 1806) calls the ‘epistemic baggage of the exploratory tradition’ – being perceived more as an adventurer than a scientist by mid-twentieth century standards. In his autobiography, Siffre recalled how he struggled to get the scientific establishment to take his work seriously, although he would go on to co-publish with leaders in the field of rhythms research (Reinberg et al., 1966).

It would be remiss to speak of cave experiments and not include the work of Manchester-based physiologist John Mills (1914–1977). Mills’ cave experiments are not as widely remarked upon as either Kleitman’s or Siffre’s, perhaps because Mills’ work was not covered extensively in the media. Rising to prominence in the 1960s, Mills was a part of a more established community of circadian researchers, participating in the founding of the Society of Biological Rhythms. A medical doctor by training, following the Second World War he turned to physiological research – focusing on renal physiology with a special interest in shift work and jet travel (*BMJ*, 1977). Despite working in the later period, Mills was still interested in that core question which troubled Kleitman – whether the human circadian rhythm was endogenous or a response to environmental changes. To answer this question, a human subject would need to be studied under ‘constant conditions’ which he observed in 1966 ‘are almost unattainable’ (p.131) in a laboratory setting. Before Mills (1978) had access to his own bunker-like purpose-built laboratory at a disused nuclear power station at Risley, he also looked underground to find a timeless experimental space.

Mills was drawn to the cave serendipitously. Unlike Kleitman and Siffre who carefully constructed (and took part in) their own experiments, Mills’ work ‘piggy backed’ on a record-setting attempt from a local man named Geoffrey Workman, who entered Stump Cross Cavern in the Yorkshire Dales on 16 June 1963 and remained there for 105 days. Workman’s explicit aim was to beat Michel Siffre’s record for a stay underground (Daily Herald, 1963). ‘I am old fashioned and patriotic enough to believe that world records should be held in Britain,’ he told the press (Freud, 1963, p. 6). Workman’s stunt became a tourist attraction for the Cavern, which like Mammoth Cave, was easily accessible from the surface. Upon hearing of his plans, Mills approached Workman to see if he could study his renal excretion patterns – and he agreed. While Workman spent most of his time exploring the cave and taking care of tasks like cooking, he also took frequent urine samples, which he placed at a pre-planned collection spot where University of Manchester scientists could take them away for analysis without interrupting his total social isolation. Otherwise, Workman had a standard 24-h watch and remained on a fairly regular schedule of sleeping, waking and eating. After emerging from the cave, he was brought to the Metabolic Ward at Manchester Royal Infirmary to undergo six-hourly blood samples and urine analysis.

Later reflecting on the experiment, Mills was critical about whether the experimental protocol was sufficiently rigorous to reveal anything meaningful about underlying rhythmic mechanisms. The subject knew the external time and lived on a standard 24-h day, despite not seeing the sun. In the later hospitalization period, meant to act as a more controlled extension of the experiment, Workman was exposed to the day/night cycle because ‘no dark curtains were available’ at the hospital (Mills, 1964, p. 218). Surrounded by such time cues, it was unlikely that he would free-run or diverge from a standard diurnal day. Nevertheless, what Mills identified in Workman’s disordered renal rhythms a ‘phase shift’. Over the course of his stay in the cave, the subject’s daily rhythmic period shifted relative to the solar day as he began to sleep and rise at later hours, while his renal metabolites lost their 24-h rhythmicity. Mills (1964, p. 229) argued that the experiment showed that some clock mechanism must control both sleep and renal excretion – but that such a clock was not exactly 24-h, permitting the observed ‘drift’.

The cave as a chronobiological field site is something quite different to the caves in other field studies. It is not, as Robert Kohler (2002a) has explored, an original place where scientists can seek the origins of evolution. These caves are also not field labs in Heggie’s (2016) conception as almost no analysis occurred in these spaces. Instead, the cave served more as a site of data collection to be taken away and processed elsewhere, in a Chicago laboratory, or a Manchester hospital. The chronobiological cave should instead be understood as a ‘mimetic place’– a space which in itself embodies the principles of the science, becoming an instrument of experimentation in itself. The characteristics of the place so prized by these researchers reflect contemporary understandings of biological time as at once social and environmental. Here we see the scientists attempting to isolate ‘time cues’ – from the more obvious (the solar light-dark cycle) to the subtle (changes in humidity). A ‘timeless’ space was one in which all measurable environmental factors are as constant as possible. Cave experiments demonstrate the importance placed by mid-twentieth century scientists on the *social* as time cue. Indeed, into the 1970s and 1980s many circadian scientists argued that social cues were among the most important zeitgebers (Minors & Waterhouse, 1981, p. 299). Caves were crucially seen as away from civilization broadly defined – from noise, from radio caves, from the rhythms of daily life and work. In all of these experiments, limiting contact with the outside world, through food deliveries, urine collection, or access to radios and time pieces, was a key element of the experimental design.

## The midnight sun

Writing in his classic textbook *Sleep and Wakefulness*, Kleitman (1939, p. 259) recalled what led him to undertake his study in Mammoth Cave. ‘Not being in a position to go to the Arctic for such an experiment, we were fortunate enough in making arrangements… to occupy a large chamber in Mammoth Cave, Kentucky.’ Although the cave experiment came to define Kleitman’s career and brought him international recognition, he dreamed of a day when he would be able to experiment with daily rhythms in an Arctic context. Mammoth Cave was a well-established tourist site – Kleitman and Richardson ‘camping’ only a short distance away from the tourist path. However, as this quote indicates, Arctic research was quite another matter – representing a significant financial and logistical investment which, in the 1930s, was beyond Kleitman’s means. While caves and Arctic spaces might be polar opposites (one dark, one light, one solitary, one social), they were perceived by physiologists interested in rhythms as two sides of the same coin – both natural laboratories for viewing the human body outside of time. Caves were relatively accessible and affordable research locations, while the Arctic was understood to be a more rigorous and informative research environment – the restricted access to which (geographically and financially) made it a pipe dream for many researchers. Here, I want to explore how early rhythm researchers conceived of the Norwegian High Arctic as a ‘timeless’ space – a field site controlled enough to rival or even surpass the findings possible in a cave or a laboratory. However, the Arctic also presented its own unique logistical and experimental challenges – ultimately proving to be not quite the perfectly timeless space some scientists had imagined.

The notion that the Arctic is a space where time stands still is a very old one. Early visitors to the region often remarked upon the physical and psychological disruption presented by the severe seasonal changes in light above the Arctic circle. In their essay on the polar summer as a ‘natural laboratory for human circadian rhythm studies’, chronobiologists Hugh Simpson and Franz Halberg quoted from the diary of the Soviet North Pole Expedition in 1937 in which the explorers ‘lost all consciousness of time’ (Simpson et al., 1973, p. 297). Kleitman’s Mammoth Cave experiment was itself inspired by a 1907 Danish overwintering expedition to Greenland where the ship’s doctor attempted to invert the body temperature curves of the crew (1939, p. 259). Achieving conditions of constant light and temperature was something that very few laboratories had an ability to recreate in the middle part of the twentieth century – making the High Arctic a uniquely potent context for disrupting and modifying daily rhythms.

Also unlike a cave space, which was generally small and confined, the Arctic was expansive – large enough for experiments to be comprised of groups of people, rather than one or two individuals. As Kleitman wrote to a correspondent, his Mammoth Cave work had the drawback of being an ‘unnatural mode of living’ – that is to say, a very small group of participants living in isolation from others (Kleitman, 1946). By contrast, the Arctic was full of people, whether scientists, explorers or indigenous peoples who, Halberg and collaborators asserted ‘are among the potentially most fruitful contributors to chronobiology’ (Simpson et al., 1970, p. 147). The notion of the ‘timeless’ Arctic in this period should also be seen in the context of wider scientific and anthropological discourse around the study of rapidly vanishing ‘primitive’ peoples in the Cold War era (Link, 2018; Radin, 2017). Physiologists were interested in timelessness in the sense of a lack of environmental time cues, as opposed to anthropology’s interest in isolated human societies as ‘timeless’ relics of the past. Nevertheless, there is a remarkable similarity between the attitudes of Kleitman, Halberg and others rhythms researchers to the way physicians and scientists in the nineteenth and twentieth centuries were attracted to studying ‘exotic’ peoples and environments. The extraordinary nature of Arctic spaces and their largely isolated populations made them desirable contexts (‘natural laboratories’) for biomedical research into acclimatisation, immunology, and, of course, rhythm studies (Farish, 2010, 2013; Heggie, 2016; Radin, 2017).

Of the early rhythms experiments in the High Arctic, among the most remarkable and overlooked by historians were carried out by pioneering physiologist and veterinarian Mary C. Lobban (1922–1982), then a researcher at the University of Cambridge’s Physiology Department. In the summers of 1953, 1955, and 1959, Lobban led the ‘Cambridge Physiological Expedition’ to the Norwegian island of Spitsbergen (Svalbard) where she and a group of students lived on modified time regimes in order to analyse the adaptability of urinary rhythms to a non-24 h day (Lobban, 1954; Lewis & Lobban, 1957a, 1957b). Like Mills, Lobban viewed urinary rhythms as a useful biological sign to assess the internal rhythmicity of the human body. While Lobban had already been carrying out studies into the modifiability of human rhythms in Cambridge, she craved a research site which could be used for larger, more ambitious experiments. As Lobban wrote in an early brief about her proposed research on the Expedition, she was seeking a place, ‘where sociological and climatological considerations would force the subjects to spend all their time in an isolated, artificially lighted room. This would, however, be much easier to obtain in some unpopulated region where light and temperature differences were minimal’ (Lobban, c.^1952^). As Lobban reflected, by the 1950s there had been some attempts to modify human circadian rhythms in laboratory settings, but this was limited to small, uncomfortable spaces which limited the length of time a study could be performed. The controlled conditions Lobban dreamed of, she asserted, were to be found ‘within the Arctic Circle during the summer months’ (Lobban, c.^1952^, ^1962^).

**Fig. 2:**
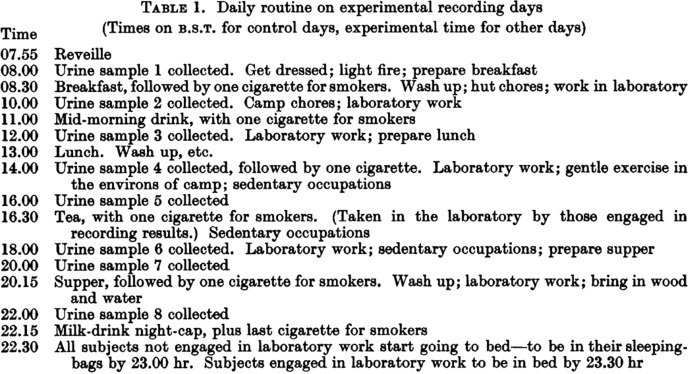
Daily routine on experimental recording days for the Cambridge Physiological Expedition, 1953. Reproduced from Lewis et al. (1956), ‘Patterns of urine flow in human subjects living on a prolonged period of life on a 22-hour day'. Courtesy of the *Journey of Physiology* (John Wiley and Sons)

The constant daylight of the Arctic summer was central to Lobban’s experimental design, which involved her human ‘guinea pigs’ living in small groups on a modified 22-h day, and in subsequent years, a 21- and a 27-h day (Lobban, 1954). The experimental time schedule was set in advance by Lobban – with ‘recording days’ requiring an exacting routine of measuring food, drink and activity, interspersed by eight urine sample collections (Fig. 2). Urine samples had the benefit of being relatively achievable measures of biological rhythmicity to take in the context of an Arctic expedition – where taking rectal temperature was highly impractical due to cold weather clothing, and blood samples might be damaged by the low temperatures. Lobban and her team of researchers also served as the experimental subjects, joined by a number Cambridge students with other scientific interests – who might sledge, survey, and sketch over the course of their two-month summer stay. To ensure experimental time was followed by all participants, Lobban arranged for the team to be provided with specially designed wrist watches by Rolex, modified to mark the altered day length (Lobban and Lewis, 1967, p. 370). The watches were especially useful in facilitating ‘strict adherence to the experimental routine’ even as participants left the base for sledging or other activities away from camp (Lobban and Lewis, 1967, p. 358).

The impulse to study the periodicity of urinary rhythms in the context of the Arctic summer is, as we have seen, far from unique to Lobban. However, how she was able to carry out these ambitious Arctic experiments is informative of the ways in which physiologists began to participate in Arctic field work and of some of the more specific challenges facing Lobban as a woman scientist in the mid-twentieth century. Access to Spitsbergen was mediated by the Cambridge geologist Brian Harland – a well-known expert on the area and himself the organizer of the Cambridge Spitsbergen Geological Expeditions, which occurred throughout the mid-twentieth century. Lobban began by approaching the Scott Polar Research Institute (SPRI) for advice on logistics and access to Spitsbergen and was directed to Harland. In private correspondence, Harland and SPRI staff initially treated her interest in Arctic fieldwork with suspicion (Unknown author, 1952). Ultimately, Lobban was able to convince Harland of her seriousness as a scientific investigator and was able to take advantage of his extensive connections and existing logistical agreements. She was even able to persuade Harland and his geology students to act as controls in her experiment (Harland, 1953). Yet her tenuous place as a woman scientist in the Arctic was not forgotten. As one participant from the 1953 Geological Expedition recalled in a personal communication, ‘Yes, we regarded it [the Physiological Expedition] as a joke. We had thought we were serious explorers, we thought of them as dilettantes. Or you know that they were bogus explorers’ (Macdonald, personal communication, July 4, 2022). Here, the participant reflected both on a disciplinary schism between geologists and physiology, but also the gender of the leader of the expedition – who had not worked in the Arctic previously.

If the Arctic space was hostile for a woman scientist, it was also not as uniform as Lobban had expected. Lobban carefully tracked fluctuations in temperature and light levels at the base camp, and while ‘no periods of darkness or of dusk were experienced throughout the experiment’, day and night were still distinguishable (Lewis et al., 1956, p. 660). Luckily, in the later 1955 expedition, the weather was largely cloudy – so that ‘the sun could not be seen and no indication was given of the real time of day’ – satisfying Lobban that in her second attempt the ‘environmental conditions were even more favourable’ than in 1953 (Lewis & Lobban, 1957a, 1957b, p. 359). Lobban also worried about the possible influence of heavy exercise (like sledging) on the experiment’s results as she was only able to limit physical activity on recording days, outside of which other scientific work like surveying needed to be done (Lewis et al., 1956, p. 660). Like Kleitman, Lobban and her collaborators concluded that the 24-h body clock was fairly flexible and that biological timing could indeed be influenced by changes in the external environment and physical activity (Lobban, 1959). While studying polar travellers and temporary visitors might answer questions about human rhythmic plasticity in the short term, Lobban was also keenly interested in studying Arctic residents (both European and indigenous) as a way to unravel longer term adaptation to photoperiods at different latitudes and in different seasons (Lobban, 1965; Lobban, 1967, 1973; Simpson & Lobban, 1967). For example, in one study, she framed her British ‘imported’ subjects from the Expedition studies as controls against which to assess relative amplitude of urinary rhythmicity in indigenous Arctic peoples (Lobban, 1967). She would go on to channel her Arctic work into shift work studies for the British Medical Research Council (MRC) as well as continuing to study Inuit communities in the context of rhythms and health (Medical Research Council, Railway Research, 1961; Lobban, 1977).

While Mammoth Cave had served as a substitute in the 1930s, as Kleitman’s renown grew, he was ultimately able to finance an Arctic experiment for himself – bringing his family with him to the Norwegian city of Tromsø in the summer of 1951 to serve as his research subjects. Kleitman secured funding for himself, his wife, and his two teenage daughters to spend nine weeks in Tromsø from May to July of 1951, during which time they would experiment with adapting themselves to a short and long day cycle. While the cave work was carried out in ‘complete isolation from community life’ – the Norwegian Arctic offered the opportunity to remove researchers from the light-dark cycle (like a cave) but to maintain ‘community conditions of living’ (Kleitman, 1950c). Modifying the environmental time cues of light and temperature while maintaining a connection to the rhythms of society was essential to furthering Kleitman’s thesis that the body’s 24-h rhythms were the result of ‘acculturation’ (Kleitman, 1964, p. 171). One may wonder why Kleitman selected Tromsø as opposed to somewhere geographically closer to his Chicago home like the North American Arctic – itself an active site of polar physiological research in this period (Farish, 2013). But, as Kleitman (1948) reflected in a letter to his Norwegian hosts, ‘it is much colder in Alaska, and I do not wish to subject my family to unavoidable hardships.’

The field site in this context was also unusual in that it was not an Arctic hut but rather a home – specifically, Kleitman and his family shared the living quarters of Sigurd Winther Hansen and his family. Hansen managed the Weather Forecasting Establishment of Northern Norway just outside downtown Tromsø – living in rooms above the weather station. This building suited Kleitman’s purposes almost perfectly. As he outlined in his proposal to Hansen, he needed a space out of town, quiet enough to avoid traffic noise, but not so far that the family could not get to town during their waking hours (Kleitman, 1948). The Kleitmans needed their own space for sleeping and preparing food, where they would not be disturbed on their modified day schedule. The weather station was also uniquely placed to provide them with round-the-clock power – electricity being rationed overnight in post-war Norway (N. Kleitman & H. Kleitman, 1953, p. 350). During their three-week experimental cycles, first an 18-h day and later a 28-h day, the family woke, ate and slept on a strict schedule – taking oral temperature readings every three to four hours, measuring heart rate, and recording diaries of their personal reflections and experiences (Kleitman, 1951). In their three-hour ‘off’ times the group would cook, do chores, play cards, or even take a trip to the local cinema.

For the Kleitmans, the Arctic was ultimately a disappointing research environment which, they remarked, ‘fell short of expectations’ (N. Kleitman & E. Kleitman, 1953, p. 290). In practice, it was easy to tell day from night as the sun moved around the house. Sunny and cloudy days also presented substantially different temperature and light environments. Kleitman later judged that the changes in temperature between night and day were ‘the greatest handicap’ to physiological stability (Kleitman & Kleitman 1953a, p. 285). Despite it’s out of the way location, the station was far from quiet. The work of the meteorologists was carried out at all hours – including checking of weather equipment on the roof above where the Kleitmans were trying to sleep. Domestic noise also filtered up through the floor from the Hansen’s residence – the sounds of children playing, a radio broadcast, or a delivery truck. The town too continued in a 24-h fashion – depending on the experimental cycle, it became impossible to shop for food or watch arrivals in the harbour. In short, Tromsø was anything but timeless – a fact reinforced by the research findings. Carrying out a survey of Tromsø inhabitants, Kleitman came to the conclusion that the sleeping habits of High Arctic residents were little affected by the seasonal changes in light – resembling the cultural practices of most Norwegians (Kleitman & Kleitman, 1953b).^4^

Like caves, the High Arctic offered a (supposedly) constant level of light and temperature, while remaining isolated from the 24-h rhythm of ‘civilized’ society in a way that would have been challenging to replicate in a contemporary laboratory setting.^5^ In practice, scientists found that the Arctic was not as controlled an environment as a cave, with notable fluctuations in light levels and temperature between day and night. At the same time, an Arctic experiment offered something closer to ‘free living conditions’ for its participants – where the experimental subjects could pursue their own work and leisure activities and for significantly longer periods of time than in either a cave or a laboratory. The length of time that it was possible to maintain the modified time regimes was a constant point of scientific discussion and a notable benefit of Arctic work. As Lobban asserted when proposing her Spitsbergen experiments, previous studies of short duration carried out in labs and even caves must be supplemented with much longer term analyses lasting several months (Lobban, c.^1952^). When it came to assessing the environmentally-influenced nature of rhythmicity, the longer the duration of the experiment the better. It is important to bear in mind that unlike animal experiments, scientists were constantly mindful of the physical and psychological discomfort of human research subjects – and it is hardly surprising that much of this challenging work was in fact self-experimentation by the investigators themselves. Despite these practical draw backs, Lobban, Kleitman and others continued to insist that the Arctic was nevertheless a useful site for rhythmic research – particularly when compared to the kind of inquiries that could happen in the cramped conditions of a cave or a laboratory.

## Cold War spaces

Thus far I have argued that caves and Arctic spaces were coveted by physiologists for their ability to isolate and modify the body’s circadian rhythms. This perceived timelessness was linked to contemporary understandings of the potential environmental (solar light-dark cycle and temperature) and social influences (family life and working patterns) on biological rhythmicity. However, subterranean caves and the High Arctic carried with them cultural, political, and even military significance beyond their usefulness as places of circadian science. As the Second World War ended and the Cold War dawned, these spaces gained cachet as analogues for the imagined futures of the atomic age – nuclear fallout, Arctic warfare, and space travel. As environmental historians of the Cold War have observed, different areas of the biosphere were intimately involved in the conflict – with the need to be constantly prepared for disaster driving new exploration and interest in the conditions of strategic natural spaces like the Poles (McNeil & Unger, 2010, p. 3). Science was a central part of the military enterprise – with scientists and military organizations entering into mutual beneficial relationships, including the provision of extensive funding and the development of new infrastructures. Many rhythms scientists were keen to exploit a new military interest in their work and were quick to highlight these connections in their grant applications and published reports, and in so doing worked to raise the prestige of rhythms research on the world stage.

As Kleitman and Richardson entered Mammoth Cave in the 1930s, the societal relevance of such experiments beyond understanding the nature of sleep was not well articulated. For most of his career, Kleitman emphasized the importance of his research as challenging outdated ideas about sleep – both within the scientific community and the public realm. In the significant press coverage surrounding the Mammoth Cave experiment, there was little mention of *why* exactly this experiment was being done – aside from pure scientific interest. As a *New York Times* correspondent reflected, ‘The practical significance [of the experiment] is rather remote’, before going on to suggest that perhaps one day in the distant future the Earth’s day length might change, making such studies of 24-h rhythms more pertinent (*New York Times*, 1938). However, in just a few years’ time with the opening of the Second World War, Kleitman and other rhythms researchers gained a new language for articulating the societal relevance of their work as sleep and preparedness became key strategic issues for industry and the military. By the 1950s, Kleitman already had significant experience as a military consultant working on optimizing shift patterns and combat readiness for soldiers, based in large part on his Mammoth Cave research (Kleitman, 1942; Wolf-Meyer, 2013).

A consummate science communicator, Kleitman was quick to adopt the concerns of the atomic age into the framing of his research on sleep and wakefulness – particularly in the Arctic context. While the High Arctic was not the location of any active fighting in the Cold War, the potential threat of Soviet dominance in the North Pole region, as well as persistent nuclear testing in the Arctic sea, gave this region military significance for European and American powers. The European High Arctic was also a particularly fractious context – given its geographical closeness with Russian territory. The experiments outlined here all benefited from the political and legal protection afforded by the Norwegian state – receiving grants to travel to the area and permission to stay at huts from the Norwegian Polar Institute and the Store Norske Spitsbergen Kulkompani – a mining company founded in 1916 which in the mid-twentieth century was a key player in logistics and shipping in the Spitsbergen area. While the surviving field notes and publications from the experiments surveyed here do not make reference to dangerous political encounters in the area, Kleitman in his correspondence arranging the Tromsø trip worried that a potential outbreak of Arctic warfare would push his experiments further and further into the future (Kleitman, 1950a, 1950b, 1950c).

For his journey to Tromsø, Kleitman applied (unsuccessfully) for funding under the 1948 – a congressional initiative that sought to counterbalance Soviet propaganda with the US government’s own informational programmes. Kleitman argued that research into sleep and wakefulness in the Arctic context ‘may prove to be of value for National Defence, whether Arctic warfare is engaged’ as it related to efficiency, alertness, and adaptability of persons to new schedules of sleeping and waking (Kleitman, 1950a). He was eventually able to obtain a small grant from the non-military Wallace C. and Clara A. Abbot Memorial Fund to support the trip (N. Kleitman & H. Kleitman, 1953). Summarizing the findings of their Norway work, Kleitman and his daughter asserted that, ‘Should the exigencies of the atomic age demand the establishment of subterranean communities, with a release from the necessary current adherence to a diurnal routine of living, an artificial cycle longer than 24-h might be preferable to a shorter one’ (N. Kleitman & E. Kleitman, 1953, p. 291). Despite his hopes of perhaps repeating the military interest that accompanied his cave work in the context of the Second World War, his attempts to use the Arctic as the basis for further defence funding was ultimately unsuccessful.^6^ Here the cave and the Arctic blend into one another – with Arctic research supporting potential new research regimes for an imagined subterranean society. Caves of course were themselves an important infrastructure in the Cold War era – serving as natural fallout shelters, alongside man-made ones like bunkers (Pike, 2021).

In his book reflecting on his experiences in Scarasson, Siffre (1965) made explicit why it was that his cave experiments had a particular relevance in the contemporary moment. Like Kleitman, Siffre was a master of obtaining his own funding – and by his own account, had only managed to secure enough financial backing for his cave experiment by begging from commercial companies, the military, and scientific organizations alike. We can imagine that the arguments he made to these groups are much like those he outlined in *Beyond Time’s* (1965) opening chapter. As we have seen, the central goal of the cave experiment was to test the limits of human endurance. But the ability to adapt to isolation had a more pressing societal relevance for Siffre:

‘This adaptive power is important in an epoch when the domestication of the atom with its consequent powers of destruction may oblige mankind to take refuge in underground shelters. It is also important to the dawning age of space travel, when individuals will be obliged to adapt themselves to a changeless environment, deprived of any points of reference in time and space’ (Siffre, 1965, p. 19).

The potential usefulness of the cave as an analogue for life in space was especially intriguing for Siffre. He wondered, ‘What kind of sleep will he [the astronaut] have, and how will his organism react to these new conditions of sleep?’ (Siffre, 1965, p. 26) Space travel, as well as the emerging age of jet travel, became a constant refrain for circadian scientists keen to emphasize the relevance of their work. NASA would go on to use insights from Siffre’s cave experiments as a part of the development of algorithms for space travel and would even sponsor a later cave experiment with Siffre in Texas (Foer & Siffre, 2008).

In his statement opening the Cold Spring Harbor Symposium, German biologist Erwin Bünning (1906–1990) observed that the scientific study of biological rhythms was important to the development of ‘east–west and west–east air travel, and even more, to space flight.’ (Bünning, 1960, p. 1) The Symposium itself had been funded in part by the United States Air Force (Ramji, 2008, p. 19). Unsurprisingly, the question of biological rhythms in space was deeply interesting to space agencies– and from this period, these organizations became key funders of circadian research. Today, both NASA and the ESA continue to use caves for the training of astronauts – both because of the physical challenges inherent in caving, but also because of their ‘timeless’ darkness. As the ESA states in its information about its CAVES programme, ‘the cave environment provides many space-relevant conditions, including isolation from the outside world, lack of diurnal cycles, confinement, minimal privacy, technical challenges, limited equipment and supplies for hygiene and comfort, and the constant presence of risk.’ (European Space Agency, 2022)

By the year 1970, the potential usefulness of rhythms research in the Cold War era had become a frequent refrain in the growing public and scientific interest in circadian rhythms. In their classic textbook on human circadian rhythms, R. T. W. Conroy and John Mills (1970, p. 6), suggested that as the result of the technological advances of the twentieth century, circadian questions were becoming more and more relevant: ‘Apart from Arctic dwellers, men have only escaped from this pervasive 24-h period by staying underground for long spells, or on submarine cruises, but this will be the natural condition of space travellers who escape from the Earth’s shadow; and those who have orbited the Earth have exposed themselves to a remarkably different period, a day of some 1 ½ hours.’ As present day circadian scientist Professor Josephine Arendt (personal communication, February 28, 2022) has observed, military and space agency funding have been a key source of grants for rhythms research since the 1960s and remain so today.

## Conclusion – Biological time between body and environment

Despite their apparent differences, in the mid-twentieth century caves and the High Arctic were frequently evoked together as natural laboratories for the study of circadian rhythms, with scientists like Kleitman moving between them. Because of their isolation both from the solar light–dark cycle and ‘society’ more generally, scientists felt that they could make use of these unique natural environments to unravel the inherent rhythmicity of biological functions like sleep, urination, and body temperature. As mid-twentieth century scientists wanted to ascertain whether the body’s 24-h biological rhythms were the result of environmental stimuli or independent of them, a successful experiment required removing a potentially unknown number of zeitgebers. Light, temperature, and sound were among the most obvious, but experimenters worried about more subtle time cues. For example, the degree to which ambient electromagnetism might serve as a time cue worried scientists – and the Andechs bunker was fitted with copper wiring to counteract its possible effects (Wever, 1979, p. 11). Biological time, it turned out, was extremely difficult if not impossible to isolate with any degree of certainty, even in a highly controlled laboratory. Some natural phenomena are simply ‘not practically amenable to experimentation’, however, as Powell (2007, p. 1797) has observed, this did little to reduce the enthusiasm of post-war scientists for ever more ambitious experimental methodologies. Despite the slippery nature of environmental and social time cues, challenging field experiments in Arctic and cave spaces seemed to offer the best possible way of modelling biological timelessness for human research subjects.

In the years around the Cold Spring Harbor Symposium on Biological Clocks in 1960, there was still considerable disagreement among rhythms researchers around the essential nature of circadian rhythms – whether they were endogenous or exogenous, whether they worked as oscillators or pendulums, and where in the body they might be located (Ramji, 2008). Cave and Arctic experiments were primarily devised to determine whether certain rhythms (like sleep) were internally or externally driven by removing those time cues that had been well established to impact rhythms – notably the light-dark cycle and ambient temperature fluctuations. It is important to re-iterate that while caves and the High Arctic are very distinctive for their photic environments, this was not the only characteristic that made them appealing as a ‘timeless’ space. Their isolation from societal rhythms was just as highly valued by the researchers like Kleitman and Lobban – following in a longer tradition of scientists fleeing from the noise and disruption of modern life (Heggie, 2016, p. 814). This fact is easy to overlook in the light of contemporary chronobiological research, which has soundly established light as the strongest zeitgeber (Czeisler et al., 1986). In this earlier context, the physical location of the research, whether cave or Arctic hut, became an essential tool to isolate and interrogate the exchanges between body and surroundings – and how this relational interactivity influenced physiological timing. If the internal rhythm of a human subject is the epistemic object of enquiry – the cave or the Arctic environment becomes a crucial ‘technical object’ in which to reveal these rhythms (Rheinberger, 2016). These are truly ‘mimetic places’ – not just background contexts, but active tools of scientific enquiry, which allowed the scientists to bring into perspective the intertwined nature of body and environments.

Whether in the cave or the Arctic, experiments into the nature of human circadian rhythms relied on an understanding of biological time as something that lies between the body and its environment. In this context, it is helpful to think about mid-twentieth century perceptions of biological timing in terms of ‘environmentality’ (Formosinho et al., 2022). This term has recently been proposed by researchers working in the context of the microbiome – arguing that recent advances in microbiome research challenge us to think past dualistic categories like inside/outside or self/other, and instead to view the environment as something that is relative, perspectival, and contextual. As Joana Formosinho and colleagues argue (2022, p. 149), there is a need to think more carefully about what is meant by the term ‘environment’. Thinking in terms of ‘environmentality’ encourages the analysis of particular, local, and temporally-specific environments. While this notion was developed to think about the concept of the holobiont (the human as microbial community), it might equally well apply to biological time as something that exists beyond the dichotomy of experimental subject and research environment. Biological timing can thus be seen as ‘situated’ – the result of a dialogue between places, objects, people and ideas (Niewöhner & Lock, 2018). This situated way of understanding body in temporal context was notably different than emerging dominant narratives within twentieth-century physiology centred on homeostasis, which imagined the body as internally self-regulating (Sellers, 2018; Simpson, 1971). Similar to Hannah Landecker’s work (2017) historicizing the closely related concept of metabolism, thinking about biological time highlights rhythmicity as undermining the boundary between organism and environment. An analysis of scientific conceptions of human circadian rhythms in specific spatial contexts provides a much needed addition to scholarship on the subject of biological timing – which thus far has tended to focus on broader timescales like that of evolution and reproduction (Rheinberger, 2002; Wellmann, 2017; Hopwood et al., 2018; Hopwood et al., 2021). Attending to biological time on the level of daily embodied experience requires tool that help us think with bodies as well as their milieus – and the temporally and spatially specific ways that scientists viewed these interactions.

It is challenging to think about field experiments in the context of their laboratory counterparts, because before the middle part of the 1960s, there were no specialist circadian laboratories. As Erwin Bünning wrote in the mid-1960s (1964, p. 1), ‘Only recently have laboratory conditions been available permitting a sufficiently accurate measurement of these physiological circadian oscillations or a satisfactory quantitative study of the influence of internal and external factors.’ Workers went to the field because there was simply no other practicable way to model the conditions they wanted to study – until considerable funding could be secured which would allow them to build facilities like the Andechs Bunker, Mills’ isolation facility at Risley, and eventually the Harvard Intensive Physiological Monitoring Unit in the later decades of the twentieth century. But these laboratories did not arise from nowhere – the principles upon which they were modelled were based on field experiments. The new ‘temporal isolation’ labs combined the environmental restrictiveness of the cave, with the greater convenience of electric lighting and plumbing – which is made it possible to isolate human subjects in relative comfort for a considerable period of time. However, these new facilities could not replicate the conditions of community living and freedom of movement and activity that came with the Arctic experiments. As a result, even as more and more specialised labs were constructed in the later part of the twentieth century, field experiments continued (and continue) to play an important role in chronobiological research – with the focus shifting towards the Antarctic from the 1980s. As Heggie (2016, p. 811) has observed, the history of science has generally neglected work done in field sites in favour of the laboratory. If this was done in the case of chronobiology, it would effectively remove the majority of human rhythms research done before 1965.

Thinking through the lens of Arctic and cave experiments also reveals the tenuous place of human chronobiological research in the middle part of the twentieth century. The experiments explored here required great resilience and persistence on the part of the investigators, who often build networks with other more established scientific disciplines. Kleitman only gained access to Mammoth Cave through introductions made for him by a senior colleague in the geology department (Kleitman, 1939b). Mary Lobban’s Cambridge Physiological Expeditions relied on the expertise and support of Brian Harland’s Geological Expeditions. While the Cold Spring Harbor meeting is generally heralded as the start of the professional scientific community, chronobiologists struggled to establish themselves as a discrete and respected discipline of the life sciences throughout the twentieth century (Cambrosio & Keating, 1983). Cave and Arctic experiments helped to establish the potential relevance of rhythms research on a wider scale – attracting critical funding from military and space sources and generating public interest in connection with the anxieties of the Cold War from space flight to nuclear fallout. By 1979, pioneering German chronobiologist Rütger Wever (1979, p. v) was confident enough to state, ‘As a consequence of these efforts, the study of biological, and, in particular, circadian rhythmicity is no longer a somewhat dubious occupation but rather a serious branch of science which combined the interdisciplinary efforts of numerous researchers around the world.’ Wever’s comments are prescient as biological timing and human health, especially in relation to shift work, jet lag, and light pollution, became the subject of increasing scientific and societal attention in the last decades of the twentieth century and into the twenty-first (Williams et al., 2021).

## Footnotes

1 This paper will not deal directly with Rütger Wever and Jürgen Aschoff’s bunker in Andechs. Although not originally built as a laboratory, it was retrofitted with scientific equipment that allowed recording and analysis to occur within its spaces.

2 This paper limits itself to human circadian experiments, although there were many prominent ‘forced desychrony’ experiments on insect, plant and animal subjects which (due to their significantly smaller scale) occurred in laboratory settings both before and contemporary to the experiments described in this paper. For a review see Kleitman (1949).

3 The prevalence of the field is clearer in historical studies of research into plant and animal rhythms. See Shackelford 2020, 2022b.

4 This sociological research diverged considerably from Kleitman’s own background as a lab scientist, and consisted of hour-long structured interviews carried out by a Norwegian sociologist and the data later analyzed by the staff of the National Opinion Research Center.

5 Kleitman, like most rhythms researchers and indeed many scientists in this period, treated the Arctic as an essentially unpopulated space, despite significant populations of indigenous peoples and resident Europeans.

6 Although it should be noted that his broader work on sleep, periodicity and efficiency quickly made its way into literature related to space flight (United States Air Force (USAF) School of Aviation Medicine, 1961).
